# Paf1c defects challenge the robustness of flower meristem termination in *Arabidopsis thaliana*

**DOI:** 10.1242/dev.173377

**Published:** 2019-10-15

**Authors:** Kateryna Fal, Matthieu Cortes, Mengying Liu, Sam Collaudin, Pradeep Das, Olivier Hamant, Christophe Trehin

**Affiliations:** Laboratoire de Reproduction et Développement des Plantes, Université de Lyon, UCB Lyon 1, ENS de Lyon, INRA, CNRS, 46 Allée d'Italie, 69364 Lyon Cedex 07, France

**Keywords:** Floral determinacy, Transcriptional noise, Paf1 complex, *WUSCHEL*, *AGAMOUS*, Carpel, Stem cell, Variability, Developmental robustness

## Abstract

Although accumulating evidence suggests that gene regulation is highly stochastic, genetic screens have successfully uncovered master developmental regulators, questioning the relationship between transcriptional noise and intrinsic robustness of development. To identify developmental modules that are more or less resilient to large-scale genetic perturbations, we used the *Arabidopsis* polymerase II-associated factor 1 complex (Paf1c) mutant *vip3*, which is impaired in several RNA polymerase II-dependent transcriptional processes. We found that the control of flower termination was not as robust as classically pictured. In angiosperms, the floral female organs, called carpels, display determinate growth: their development requires the arrest of stem cell maintenance. In *vip3* mutant flowers, carpels displayed a highly variable morphology, with different degrees of indeterminacy defects up to wild-type size inflorescence emerging from carpels. This phenotype was associated with variable expression of two key regulators of flower termination and stem cell maintenance in flowers, *WUSCHEL* and *AGAMOUS*. The phenotype was also dependent on growth conditions. Together, these results highlight the surprisingly plastic nature of stem cell maintenance in plants and its dependence on Paf1c.

## Abstract (precis)

**Summary:** Using a mutant with increased transcriptional noise, we reveal that stem cell maintenance is not as robust as anticipated in plants, even leading to major defects in essential developmental processes such as flower indeterminacy.

## INTRODUCTION

Developmental robustness is ambivalent: patterns of growth must be reproducible, as body plans are usually comparable within individuals of given species; they must also be plastic to enable adaption to external and internal changes and fluctuations. In other words, developmental robustness entails a balance between homeostatic mechanisms that ensure that many phenotypes are robust to genetic and environmental variations and promotion of variability to trigger alternative developmental pathways to face genetic and environmental variations. This balance is also a variable, as the ratio between reproducibility and variability promotion can shift as development progresses (see, for example, Tsugawa et al., 2017).

Among the factors behind developmental robustness, transcriptional noise can contribute to specific differentiation pathways in various tissues (Mason et al., 2014; Mantsoki et al., 2016; Alemu et al., 2014; Padovan-Merhar and Raj, 2013; Sprinzak et al., 2010). In addition, the maintenance of stem cells might rely on the relative inefficiency of the transcriptional and translational machinery that maintains the stem cells in an indeterminate state (Momiji and Monk, 2009). Interestingly, variability of gene expression can account for reduced penetrance (Raj et al., 2010). In plants, the contribution of gene expression variability to plant developmental robustness and plasticity remains poorly documented. Gene expression variability has mainly been assessed during responses to external or internal stimuli (Waters et al., 2017; Xu et al., 2016; Wang et al., 2011) and only more recently as an internal input to support developmental plasticity at the tissue level (Meyer et al., 2017).

Although the exact mechanisms behind transcriptional noise remain to be uncovered, relevant molecular factors are starting to be identified. For instance, the variability of gene expression in mammals relies on several features of the gene itself, spanning from its genomic structure and regulation to its interacting network (Alemu et al., 2014). Interestingly, the RNA polymerase II-associated factor 1 complex (Paf1c) seems to play a key role in this process. Mutations in Paf1c subunits increase gene expression noise in yeast (Ansel et al., 2008; Richard and Yvert, 2014). This effect not only relies on the functional interaction with RNA polymerase II, but also on a larger spectrum of activities. In plants, Paf1c has been shown to influence gene expression through regulation of transcription (Oh et al., 2004; Antosz et al., 2017) and modification of chromatin (He et al., 2004; Oh et al., 2008). In mammals, Paf1c also restrains the activation of enhancers and thus hinders the release of paused RNA polymerase II, adding another layer of control of gene expression variability (Chen et al., 2017). In principle, mutations in Paf1c subunits offer the ideal context for analyzing the role of transcriptional noise in development.

One of the Paf1c components, VERNALIZATION INDEPENDENCE 3 (VIP3), was initially shown to control flowering time (Zhang et al., 2003). Recently, *vip3* mutants were found to exhibit variable phyllotactic patterns: *vip3* mutants exhibit an average divergence angle of 137° between each organ initiation at the shoot apex, as in the wild type, but the standard deviation of that angle is increased in the mutant (Fal et al., 2017). Because no other mutant exhibits such a phenotype, this finding suggests that Paf1c-dependent transcriptional control is important for developmental robustness as a whole. Here, we investigate whether flower termination, a developmental process that is both central to plant reproduction and very reproducible, also depends on Paf1c.

Flowers are produced by the shoot apical meristem (SAM), which hosts a pool of pluripotent stem cells. This explains why the SAM at the tip of an inflorescence stem produces an indeterminate number of flowers (Besnard et al., 2011). Young flowers also exhibit early meristematic activity but, in contrast to the SAM, they produce a determinate number of organs (four sepals, four petals, six stamens and two carpels in *Arabidopsis thaliana*). This implies that maintenance of the stem cell pool stops as the flower matures. Two decades of molecular genetics have demonstrated that stem cell homeostasis relies on a negative feedback loop involving the WUSCHEL (WUS) and CLAVATA (CLV) factors (Somssich et al., 2016). *WUS* encodes a homeodomain transcription factor and is expressed deep inside the SAM, in the organizing center (Mayer et al., 1998). The WUS protein moves to the central zone to promote both stem cell identity and *CLV3* expression (Yadav et al., 2011; Daum et al., 2014). The CLV3 ligand diffuses in the upper part of the meristem and triggers the CLV-CORYNE pathway that, together with RPK2, restricts *WUS* expression to the organizing center (Lenhard and Laux, 2003; Rojo et al., 2002; Kinoshita et al., 2010; Brand, 2000; Schoof et al., 2000; Müller et al., 2008). The *ERECTA* (*ER*) receptor kinase and most of the *HD-ZIPIII* genes have been shown to regulate meristem size and stem cell homeostasis through different pathways and in parallel to the CLV pathway (Green et al., 2005; Prigge et al., 2005; Williams, 2005; Mandel et al., 2014, 2016). All these genetic pathways, together with additional layers of control such as transcriptional regulators HAM (Zhou et al., 2018) and ULTRAPETALA1/2 (ULT1/2) (Carles, 2005; Monfared et al., 2013), chromatin regulators FAS1/2 (Kaya et al., 2001) and SYD (Kwon, 2005), cytokinins (Leibfried et al., 2005; Gordon et al., 2009), meristem geometry (Gruel et al., 2016) and environmental factors (Pfeiffer et al., 2017), robustly maintain and confine the stem cell niche before flowers are produced.

The flower initially inherits the potential of indeterminacy from the SAM: the maintenance of stem cells in the center of the flower relies on the same WUS/CLV regulatory loop (Schoof et al., 2000). Floral termination coincides with the end of *WUS* expression once carpels have been produced, at stage 6 (Smyth et al., 1990) in *A. thaliana* (Mayer et al., 1998). AGAMOUS (AG), a MADS box transcription factor (Yanofsky et al., 1990), is a key regulator in this process and triggers flower meristem termination by repressing *WUS* expression (Lohmann et al., 2001; Lenhard et al., 2001). This repression by AG can be direct, by recruiting polycomb group (PcG) factors and promoting a chromatin loop that blocks the recruitment of RNA polymerase II at the *WUS* locus (Liu et al., 2011; Guo et al., 2018), but also indirect through activation of KNUCKLES (KNU; a C2H2 Zn-finger transcription factor) (Sun et al., 2009). KNU is recruited to the *WUS* locus by MINI ZINC FINGER2 to form a complex together with HISTONE DEACETYLASE-like HDA19 and TOPLESS, which in turn inhibits *WUS* expression (Sun et al., 2009, 2014; Bollier et al., 2018). KNU also directly binds the *WUS* locus to cause eviction of SYD and subsequent recruitment of PcG factors to silence *WUS* (Sun et al., 2019). Consistently, most mutants showing flower termination defects also show a transient reduction in *AG* expression in the center of the flower (Clark et al., 1993; Fletcher, 2001; Prunet et al., 2008; Das et al., 2009; Maier et al., 2009). Interestingly, recent data report how AG also influences auxin and cytokinin biosynthesis during the flower meristem termination process (Yamaguchi et al., 2018; Zhang et al., 2018). Similarly, expression of a miR172-insentive version of *APETALA2* (*AP2*) results in a decrease in *AG* expression and in the development of supernumerary organs in the center of the flower (Zhao et al., 2007). AP2 may also promote floral stem cell maintenance by counteracting *AG* function (Zhao et al., 2007; Liu et al., 2014; Huang et al., 2017). Interestingly, mutations in many genes reported above as involved in the control of stem cell homeostasis in the SAM (including *CLV*, *ULT*, *ER*, *HD-ZipIII*) result in flower meristem indeterminacy, this phenotype often being related to a defect in *AG* expression. It seems, therefore, that *AG* expression is a good integrator and proxy for the final developmental decision to switch from an indeterminate to a determinate flower. Although single mutants have revealed that this process can be impaired, the contribution of transcriptional noise to the robustness of flower termination remains unknown.

We report here that mutations in Paf1c can result in loss of floral determinacy. Such a phenotype is caused by maintenance of stem cells in the center of the flower beyond stage 6, which results in a global decrease in *AG* expression in the center of the flower. Importantly, this phenotype is not fully penetrant, with flowers exhibiting subtle defects to fully indeterminate phenotypes, even on the same individual plant. This phenotype also depends on environmental conditions, suggesting that Paf1c integrates both developmental and environmental cues to reduce *AG* expression variability during flower development and to hinder floral indeterminacy.

## RESULTS

### *vip3* mutants exhibit strong and variable flower indeterminacy

**Fig. 1.**
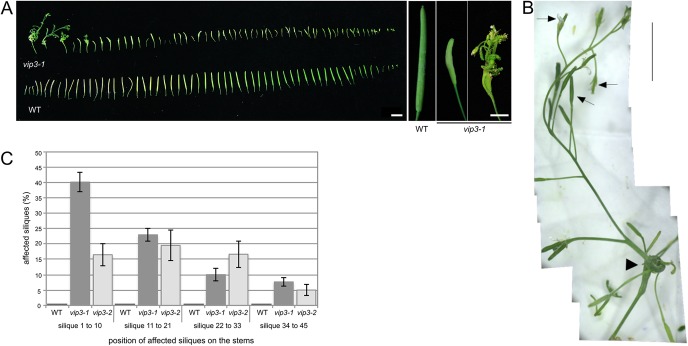
***vip3* mutants can exhibit a severe flower indeterminacy phenotype.** (A) Left: Representative phenotype of wild-type and *vip3* siliques, from plants grown in short day conditions at 21°C then continuous light at 16°C (*N*>30 plants), harvested from the stems in a sequence of initiation. Right: representative siliques of the wild type and *vip3* displaying different degrees of phenotypic defects. (B) Representative image of the most severe phenotype in *vip3-1* flowers. Arrowhead points at the primary silique; arrows point at secondary carpels. (C) Distribution (%) of affected siliques on the stems of the wild type (*N*=13), *vip3-1* (*N*=60) and *vip3-2* (*N*=20) grown in short day conditions at 21°C then continuous light at 16°C. On average, 20% of *vip3-1* and 14% of *vip3-2* siliques displayed visible indeterminacy defects in these conditions. Scale bars: 1 cm in A, left panel; 5 mm in A, right panel; 1 cm in B.

*vip3* mutants have previously been reported to display a number of growth defects (Zhang et al., 2003; Takagi and Ueguchi, 2012; Dorcey et al., 2012; Fal et al., 2017). When *vip3* mutants were grown for 3 weeks under short day conditions (see Materials and Methods) at 21°C and then transferred to continuous light at 16°C, we observed a dramatic loss of floral indeterminacy such that, in some *vip3* plants, a wild-type sized inflorescence would grow out of a carpel (*N*>30 plants, Fig. 1A,B; Fig. S1). Whereas this phenotype was observed in both *vip3-1* and *vip3-2* alleles (Fig. 1C), silique development in the wild type remained entirely unaffected under these growth conditions (*N*>30 plants, Fig. 1A,C).

To check whether this phenotype depends on either the temperature or day length shift, we next studied the *vip3-1* phenotype under different growth conditions. Plants grown in continuous light but at 21°C instead of 16°C displayed a similar phenotype (*N*=32 plants, Fig. S2). We could also see the indeterminacy phenotype when *vip3-1* was constantly grown under short day conditions (*N*=9 plants, Fig. S3A) and under short then long day conditions (*N*=22 plants, Fig. S3B). When grown in long days, the *vip3* mutant was much smaller, with shorter stems, and exhibited a large number of aborted siliques without indeterminacy (*N*=36 plants, Fig. S3C). Therefore, floral termination defects in *vip3* only require short day conditions and no other specific growth conditions. Note that the *vip3* mutant was able to produce seeds but at a very low rate (Fig. S4), except when plants were grown exclusively under long day conditions that resulted in sterile siliques (Fig. S3C).

The extent of the floral indeterminacy defects in *vip3* depended on growth conditions: the *vip3* phenotype was most affected in short day and in short day then continuous light (16°C or 21°C) conditions and appeared to be the closest to a full reversal of floral identity, as reported in the literature. Note that we observed similar phenotypic defects in *vip6*, a mutant for another component of the Paf1 complex (*N*=19 plants, Fig. S3D). Such data further confirm that flower phenotypes result from defects in the Paf1-C and not in the exome complex, which is involved in mRNA turnover and which VIP3 (SKI8 analog), but not VIP6, is part of (Dorcey et al., 2012).

Furthermore, the *vip3* indeterminacy phenotype was also highly variable within a single plant (Fig. 1A; Fig. S2A). In comparison to the wild type, the phenotype ranged from short and bumpy siliques to completely open siliques containing a full inflorescence. With respect to the position of the siliques along the inflorescence stem, we found that early siliques were very often the most affected, although even the last siliques occasionally exhibited a strong phenotype (Fig. 1C; Fig. S2B).

### Supernumerary organs develop from the center of the floral meristem

**Fig. 2.**
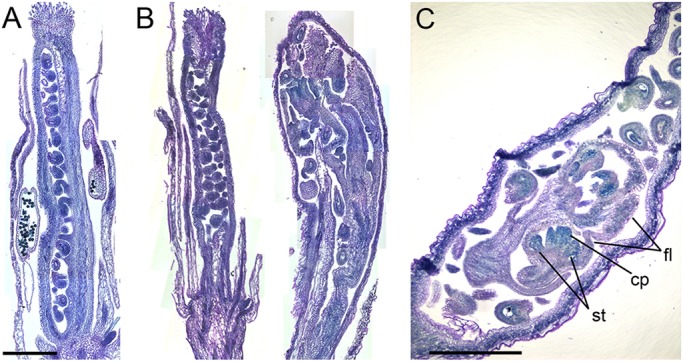
**Inflorescence stem and floral organs can be detected in *vip3* siliques.** (A,B) Sections in young siliques, stained with toluidine blue. Wild-type (A) and representative *vip3* (B) siliques illustrate the spectrum of *vip3* phenotypes. (C) Section of young *vip3* silique, stained with Toluidine Blue, demonstrating the presence of floral structures inside the silique. cp, carpel; fl, flower; st, stamen. Scale bars: 500 μm.

Except for branching meristems that develop from bract axils in species with a dichasium inflorescence (Claßen-Bockhoff and Bull-Hereñu, 2013) or from sepal axils in *ap1* mutants that lack petals and have sepals displaying bract-like features (Irish and Sussex, 1990; Mandel et al., 1992), there are two ways in which flower indeterminacy can occur: either the flower maintains its stem cells after stage 6 (Prunet et al., 2009) or ovules are homeotically converted into carpels (Modrusan et al., 1994; Pautot et al., 2001). In the latter case, one would expect to see multiple carpels growing within a single primary carpel. We never observed such a phenotype in *vip3* mutants; instead, the supernumerary organs all arose from the same stem or at least belonged to the same structure. It is therefore more likely that flower indeterminacy in *vip3* mutants is the result of a delay in flower termination. To confirm that hypothesis, we generated longitudinal sections through carpels in both wild-type and *vip3* carpels and stained the structures with toluidine blue. We observed that supernumerary organs always developed within the primary carpels on a stem emerging from the bottom of the flower (*N*=44 carpels, Fig. 2). We never detected supernumerary organs emerging from ovules. The presence of such long stems within the carpel has not been reported in other indeterminate mutants such as *crc ult*, *crc sqn*, *crc rbl*, *pwd*, *clv1* or *knu* (Prunet et al., 2008; Yumul et al., 2013; Clark et al., 1993; Sun et al., 2009).

### RNA-seq analysis of *vip3-1* mutant shoot apices reveals genomewide expression defects

Given the strength of the phenotype, we first checked whether specific pathways are affected in *vip3*. To do so, we performed RNA-seq analyses of the *vip3-1* mutant, using shoot apices (Fig. S5A,B). Note that this material only contained meristems and flowers up to stage 3 (i.e. not fully developed). The fold change for each gene was expressed in the log2 scale (meaning that a factor of 1 corresponds to a twofold change). This analysis revealed defects in *FLOWERING LOCUS C* (*FLC*) expression (downregulation by a factor 4.6; Fig. S5C), as already reported (Oh et al., 2008). However, this large-scale analysis did not reveal clear-cut defects in specific flowering pathways, but global defects in the transcriptome, even if we cannot exclude any defects on specific pathways due to statistical and/or detailed annotations limitations. Genes from the same family (e.g. MADS) displayed either reduced (e.g. *AGL31*, *AGL77*) or enhanced (e.g. *AGL71*) mRNA accumulation in *vip3-1* (Fig. S5C). A few putative regulators of *WUS*, such as *ULT2*, exhibited a significant decrease in mRNA accumulation (by a factor of 3.1), whereas *CLV3* mRNA accumulation was higher (by a factor of 2.4) in *vip3-1* (Fig. S5C)*.* Other putative regulators such as *PHB*, *ERL1*, *HAM3* and *PAN* also show higher mRNA accumulations but with lower rates (by factors of 0.6, 0.8, 1 and 1.1, respectively; Fig. S5C). Similarly, we also found that hormone signaling pathways were affected, albeit without any clear-cut, specific trend. Yet, expression of genes involved in both auxin and cytokinin pathways seemed to be affected (Fig. S5D). Such data are consistent with previously reported phyllotactic defects in *vip3* (Fal et al., 2017) and with more recent data on hormonal control of floral determinacy (Yamaguchi et al., 2018; Zhang et al., 2018) as well as with the indeterminacy defects reported here. Note that RNA-seq data obtained previously on *vip3* seedlings also reflects such genomewide alteration, without clear-cut targets (Oh et al., 2008). Together, these data are consistent with the hypothesis that the *vip3* mutant does not affect specific pathways, but instead increases transcriptional noise, as assessed in yeast (Ansel et al., 2008). Ideally, single-cell RNA-seq analyses would provide quantitative data on transcriptional noise in plants. These results thus call for gene-by-gene analysis of expression patterns of specific regulators of stem cell maintenance and flower termination.

### Development of supernumerary organs results from the prolonged maintenance of stem cells in the center of the flower

**Fig. 3.**
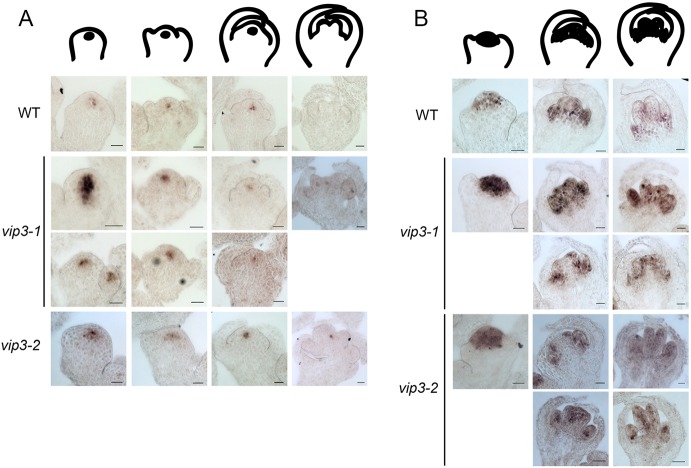
**Expression patterns of *WUS* and *AG* in *vip3* flowers.** Representative *in situ* hybridization of (A) *WUS* (*N*_WT_=34 flowers, *N_vip3-1_*=30 flowers, *N_vip3-2_*=45 flowers) and (B) *AG* (*N*_WT_=33 flowers, *N_vip3-1_*=35 flowers, *N_vip3-2_*=12 flowers) transcripts in wild-type and *vip3* (*vip3-1* and *vip3-2*) flowers at four or three different developmental stages (as represented by the drawings). Plants for hybridization were grown in short day conditions at 21°C then continuous light at 16°C (as in Fig. 1). Scale bars: 20 μm.

**Fig. 4.**
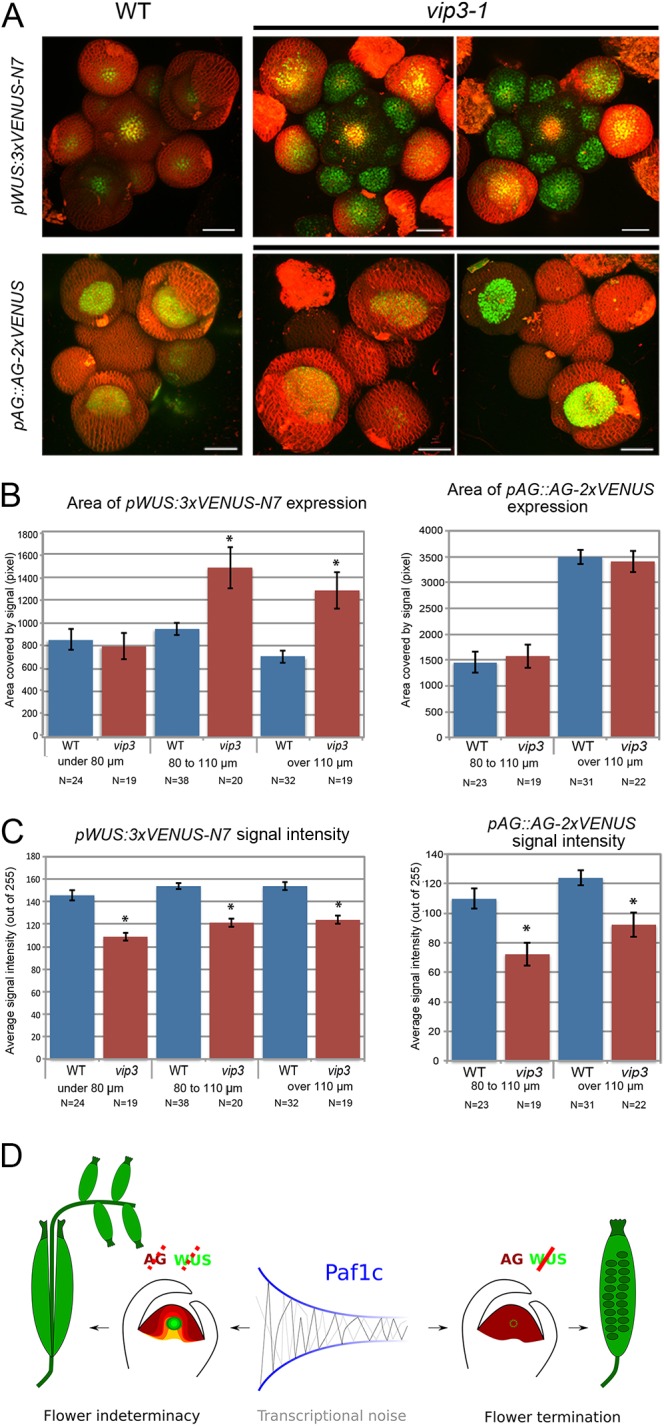
**Expression of *WUS* and *AG* reporter lines in *vip3* flowers.** (A) Representative wild-type and *vip3-1* inflorescence meristems expressing *pWUS::3xVENUS-N7* (*N*_WT_=94 flowers, *N_vip3-1_*=58 flowers) and *pAG::AG-2xVENUS* (*N*_WT_=54 flowers, *N_vip3-1_*=41 flowers) reporters, labeled with FM4-64. (B) Area of *pWUS::3xVENUS-N7* (left) and *pAG::AG-2xVENUS* (right) expression in wild-type and *vip3-1* flowers of different size (<80, 80-110 and >110 µm). Flower diameter was calculated as described in Materials and Methods. (C) Average signal intensities (mean±s.e.m.) for *pWUS::3xVENUS-N7* (left) and *pAG::AG-2xVENUS* (right) in wild-type and *vip3-1* flowers of different size (<80, 80-110 and >110 µm). Results were considered significant when *P<0.05% by the two-tailed Student’s test. (D) VIP3 contributes to the robustness of flower meristem termination. Scale bars: 50 μm in A.

Because our phenotypic analysis suggested that the *vip3* indeterminacy phenotype was caused by prolonged maintenance of stem cells in flowers, we focused our analysis on the integrator of stem cell maintenance and flower termination, *WUS*. Using *in situ* hybridization, we observed a bright and localized signal in the organizing center of wild-type SAM and young flowers until stage 5 or 6 (*N*_WT_=34 flowers; Fig. 3A) (Mayer et al., 1998). In *vip3*, we observed some flowers with a similar pattern, but others with more variable patterns. In particular, we detected *WUS* expression at the center of flowers at a much later stage than in the wild type (Fig. 3A; Fig. S6B), which is consistent with the indeterminacy phenotype. The *WUS* expression domain was also much broader than that of the wild type in certain *vip3* flowers (*N_vip3-1_*=30 flowers, *N_vip3-2_*=45 flowers; Fig. 3A; Fig. S6). To account for this variability in the spatial domain of *WUS* mRNA accumulation in *vip3*, we distinguished different types of patterns: the wild type displayed a single robust pattern, but the *vip3* mutant exhibited either a normal *WUS* expression domain (in 51 out of 73 meristems) or a larger and deeper *WUS* expression domain (in 22 out of 73 meristems; Fig. S6C). To further confirm these trends, we next analyzed the expression of *WUS* in a line expressing a fluorescent tag under the control of *WUS* promoter *pWUS::3xVENUS-N7* (Pfeiffer et al., 2016). The fluorescent pattern was wider in both wild-type and mutant flowers, as compared with our *in situ* hybridization data. Wider *pWUS::GFP* expression domains in the wild type have already been reported (Gordon et al., 2009). Nevertheless, we clearly observed an even wider expression of *WUS* in *vip3* flowers compared with wild-type flowers (*N*_WT_=94 flowers, *N_vip3-1_*=58 flowers; Fig. 4A). Quantification of the area of *WUS* expression revealed it to be up to two times larger in *vip3* than in the wild type (Fig. 4B). The coefficient of variation of *WUS* expression area was also significantly increased in *vip3* (Fig. S7A). Quantification of the average fluorescence intensity suggested a mild reduction in *WUS* promoter activity in *vip3*, although this might reflect a larger gradient domain (Fig. 4C). Based on both *in situ* hybridization data and fluorescent reporter lines, the *WUS* expression domain appeared variable and rather enlarged in *vip3*. As ectopic expression of *WUS* in flowers is also known to generate extra organs in the center of the flower (Lenhard et al., 2001), our data are consistent with the macroscopic indeterminacy phenotype in *vip3*. Note that we could not detect a significant effect of *vip3* mutation on the *CLV3* spatial expression domain by *in situ* hybridization. Yet, *CLV3* expression seemed to be maintained at later flower stages than in the wild type (in 6 out of 15 flower meristems beyond stage 6; *N*_WT_=10 meristems, 12 flowers; *N_vip3-1_*=9 meristems, 14 flowers; *N_vip3-2_*=5 meristems, 5 flowers; Fig. S8). This is consistent with an overall delay in flower termination.

### Mutation in *VIP3* results in a lower expression of AG in the center of the flower

Given that the *vip3* indeterminacy phenotype is strong and variable, and that it is associated with perturbed stem cell maintenance control, we analyzed expression of *AGAMOUS* (*AG*), the primary regulator of stem cell arrest in the flower. Analysis of the *AG* mRNA pattern through *in situ* hybridization revealed the expected pattern in the wild type, with strong accumulations in floral whorls 3 and 4, prior to the emergence of stamens and carpels (*N*_WT_=33 flowers; Fig. 3B). Similar patterns were also observed in certain *vip3* flowers, but *AG* mRNA accumulation appeared much reduced in the center of whorl 4 in other flowers (*N_vip3-1_*=35 flowers, *N_vip3-2_*=12 flowers; Fig. 3B). To further confirm this result, we generated a fluorescently tagged version of AG under its own promoter (*pAG::AG-2xVenus*) and analyzed its expression profile. These data confirmed the results from the *in situ* hybridizations, and also showed a globally reduced level of AG in certain *vip3* flowers (*N*_WT_=54 flowers, *N_vip3-1_*=41 flowers; Fig. 4A,C). AG signal intensity was also more variable in *vip3* (Fig. S7B). The global area of AG expression was not significantly different in *vip3* and the wild type, consistent with the observations that the contours were not strongly affected and that only the center of flower exhibited defects in AG expression (Fig. 4B). Together, these results show that defects in Paf1c-dependent control of transcriptional noise lead to a delay in flower termination, notably through *AG* and *WUS* (Fig. 4D).

## DISCUSSION

We have uncovered a strong floral indeterminacy phenotype in *vip3*. Flower development is usually considered to be highly robust in *A. thaliana*. Nonetheless, chimeric flowers can be produced at low frequency (Hempel and Feldman, 1995). Such flowers result from primordia exhibiting the features of both flowers and paraclades (lateral flowering shoot). Here, *vip3* flowers develop normally (in terms of identity) but a variable proportion do not stop producing organs beyond stage 6, resulting in short and bumpy siliques up to completely open siliques containing a full inflorescence. Most indeterminacy phenotypes reported so far result in the production of extra floral organs, mostly carpels and stamens, rarely petals except in strong *ag* mutants that reiterate complete flowers (Bowman et al., 1989; Prunet et al., 2009). Thus, in mutants with weaker phenotypes than that of *vip3*, floral meristem identity is never, or extremely rarely, lost. The only cases where a full new inflorescence was reported is in *clv1-4* ﬂowers where, in rare cases, a new inﬂorescence with developing ﬂowers emerges from the gynoecium (Clark et al., 1993). Although this new inflorescence is obtained through gain of function, the *p35S::XAL2* line, in which the MADS box transcription factor XAL2/AGL14 is overexpressed, also displays major indeterminacy defects that resemble those of *vip3* mutants (Pérez-Ruiz et al., 2015). Our results in *vip3* mutants suggest that full reversion might be reachable through a more global perturbation of transcription. This calls for a more systemic investigation of the molecular players behind floral indeterminacy. In fact, these results also question the limits of the reductionist approach: genetic screens for floral indeterminacy did not uncover the *vip3* mutant, either because growth conditions were not appropriate or because variable phenotypes are less likely to be identified and selected.

Early stages of growth in short day conditions appeared essential to trigger the indeterminacy phenotype in *vip3*. This is consistent with the reported role of the Paf1 complex in the regulation of flowering time and *FLC* expression (Zhang et al., 2003). The results also reveal that a late phenotype (carpel differentiation) depends on very early cues during development. Our findings thus suggest that floral indeterminacy is much more plastic than anticipated, integrating the larger plant status early in development. The indeterminacy defects are not detected in long day conditions, but are observed in short day or continuous light conditions. Given that the latter growth conditions enhance meristem size (Landrein et al., 2015), it is possible that a threshold in meristem size is required for the indeterminacy phenotype to exist. In this respect, cytokinins are likely to play a strong integrator role, given their known impact on the regulation of *WUS* expression and meristem size (Pfeiffer et al., 2016; Landrein et al., 2018). Beyond cytokinins, the larger hormonal network is probably involved. For instance, in our RNA-seq analysis, we also found that YUC4, a target of AG and CRC (Yamaguchi et al., 2018), was downregulated in *vip3-1*. It remains to be shown whether such conclusions apply to other species; data in Impatiens balsamina suggest that it is the case (Pouteau et al., 1997).

As *AG* is deregulated in *vip3* mutants, our study also introduces Paf1c as a new player in the flower termination pathway. The use of lines expressing the antisense *AG* RNA gave a range of phenotypes, spanning from a weak indeterminacy phenotype (normal flower with few extra organs developing inside the primary carpels) to the canonical *ag* phenotype ([sepals-petals-petals]*n*), each category corresponding to a lower level of endogenous *AG* expression (Mizukami and Ma, 1995). In *vip3*, we observed weaker AG expression in the floral domain that corresponds to the fourth whorl subdomain that develops carpel margins and placenta. The reduced AG level in *vip3* might be consistent with the reported increase in H3K27me3 over the *AG* region in the mutant (see figure S4 in Oh et al., 2008). Our study thus opens the possibility that part of the plasticity in carpel development relies on Paf1c-dependent *AG* expression.

Our results echo the rising role of incomplete penetrance in developmental plasticity. Incomplete penetrance is intrinsically caused by random fluctuations in gene expression (Raj and van Oudenaarden, 2008). Such variability contributes to cell fate specification in multicellular organisms (Chang et al., 2008; Hume, 2000; Wernet et al., 2006). The existence of such variability could lead to incoherencies in gene networks; yet it can also provide a way for the network to become less sensitive to environmental fluctuations. In other words, cells can still retain the ability to acquire alternative fates, despite the channeling effect of environmental cues (Hart et al., 2014). Interestingly, we find that the *vip3* indeterminacy phenotype occurs when *WUS* expression slowly decreases in wild-type flowers. Gene expression fading (in and out) and low levels of gene expression might represent weak points in gene networks, as variability in gene expression (area, intensity and duration) in such instances can have more pronounced effects. Conversely, the gene regulatory network often promotes clear-cut expression patterns (both in space and time) and this could limit the presence of such weak points. It appears surprising that a developmental switch as important as the decision to stop or maintain stem cells in a flower relies on such a robust Boolean control, yet our results in the *vip3* mutant suggest that increased transcriptional noise is sufficient to induce indeterminacy. This calls for an analysis of the adaptive benefits of such a weak control. One could speculate that the number of fruits and seeds would be increased via this unusual prolongation of floral stem cell competence, as observed in other species (Tooke et al., 2005).

## MATERIALS AND METHODS

### Plant lines

All procedures were performed on plants from the Col-0 ecotype. The *pWUS::3xVENUS-N7* reporter lines (Pfeiffer et al., 2016) and T-DNA insertion lines *vip3-1* (salk139885) and *vip3-2* (salk083364) were used for this study (genotyping primers are listed in Table S1). To generate the *pAG::AG-2xVenus* line, we used a fragment of genomic *AG* from Col-0, containing 2655 bp of upstream sequence, the 1061 bp 5′UTR (which includes intron 1) and 4241 bp from start to stop (which includes the 2999 bp second intron), amplified with the pPD381 and pPD413 primers (see Table S1) and transferred with *Xma*I digestion in BJ36 containing 2xVenus fluorescent reporter. BJ36 with 2xVenus was obtained from pCS2-Venus with pPD441 and pPD442 primers (see Table S1) adding 5xAla at the beginning of Venus and transferred twice in BJ36 through *Bam*HI and *Xma*I digestion. The *pAG::AG-2xVenus* obtained fragment was transferred in *pART* (a kanamycin-resistant vector) with *Xma*I digestion and then transformed in Col-0 plants using *Agrobacterium tumefaciens*.

### Growth conditions

In ‘short day’ conditions, plants were grown under a 8 h (21°C)/16 h (15°C) light/dark period. In ‘long day’ conditions, plants were grown under a 16 h (21°C)/8 h (19°C) light/dark period. In continuous light conditions, plants were grown under continuous light at 16°C or 21°C. In ‘short day then long day or continuous light conditions’, plants were first grown for 3 weeks in short day conditions and then transferred to long day or continuous light conditions.

### RNA-seq analysis of *vip3* shoot apices

*vip3-1* and Col-0 shoot apices (from plants grown in short day conditions at 21°C then continuous light at 16°C) were dissected by removing flowers older than stage 4. Samples were collected into liquid nitrogen-cooled Eppendorf tubes directly after dissection, each tube containing between 30 and 35 apices, 6 samples for each genotype. RNA extraction was performed using the PicoPure RNA Isolation Kit Arcturus (ThermoFisher, KIT0204) with an on-column DNase treatment (Qiagen, catalog#79254). After elution, two samples were combined together, obtaining the final technical triplicates for each genotype. RNA concentrations in the samples were measured by Bioanalyser (Plant RNA Nano Assay, Agilent Technologies, Chip priming station number 5065-4401, 16-pin bayonet electrode cartridge, order number 5065-4413) and sent for sequencing. Total RNA libraries preparation, Illumina sequencing and initial data analysis were performed by Fasteris (HiSeq instrument, Basecalling pipeline, HiSeq Control Software HD 3.4.0.38, analysed with Expression_mRNA_tuxedo). Adapter trimming was with Trimmomatic, a flexible read trimming tool for Illumina NGS data (Bolger et al., 2014). Mapping was with BOWTIE 2.0.5 (Langmead et al., 2009), TOPHAT 2.0.6 (tophat.cbcb.umd.edu/) and SAMTOOLS 1.2 (www.htslib.org/). The reference genome was *Arabidopsis thaliana* Ensembly TAIR10, from iGenome. Expression estimation, normalization and comparison was carried out using CUFFLINKS v2.1.1 (cufflinks.cbcb.umd.edu/).

### Histological sections and *in situ* hybridization

The *in situ* hybridization on paraplast-embedded tissues was performed as described (Vernoux et al., 2011). Shoot apices were sectioned into slices 8 μm thick. The probes for the coding regions of *WUS* and *AG* were amplified with specific primers (listed in Table S1), where the T7 promoter sequence was added to the reverse primer. PCR products were further purified with the QIAquick PCR Purification Kit (Qiagen ID 28106). *In vitro* transcription and DIG labeling of the probes were performed with the T7 RNA polymerase (Promega, P2077) and DIG RNA Labeling Mix (Roche 11277073910). For histological sections, late flowers (stage 15-16) were harvested and paraplast-embedded following the same protocol. After sectioning, paraffin removal and rehydration, the samples were stained with 0.1% toluidine blue solution. Images were acquired using the Zeiss Imager.M2 microscope (20× and 40× objectives) and the Axiocam 503. Results were obtained in triplicates (three independent rounds of *in situ* hybridizations, from independently grown plant populations).

### Confocal laser scanning microscopy and image analysis

Dissected meristems and plants grown *in vitro* were imaged with a water dipping lens (25×, NA=0.8) using a SP8 confocal microscope (Leica, Germany) to generate a stack of optical sections with an interval of 0.2 μm between slices. The membranes were stained with FM4-64. Image analysis was performed using the Fiji software (fiji.sc/wiki/index.php/Fiji). The fluorescence intensity and size of the fluorescent area were extracted from the maximum projections of the image stacks of each individual flowers using the ROI tool. For smaller flowers, about 280 slices were imaged, representing a stack 56 μm thick; for older flowers, about 430 slices were imaged, representing a stack 87 μm thick. Average diameter of the flowers was calculated by tracing four lines between the edges of a flower, crossing in the center with a 45° angle between each pair of lines. The extracted ROI values were further analyzed using Microsoft Excel. Statistical analysis was performed using either Microsoft Excel or R softwares. The two-tailed Student’s test was used to compare means of independent biological replicates. Results were obtained in triplicates (three independent rounds of imaging sessions, from independently grown plant populations).

## Supplementary Material

Supplementary information
