# Supplementary material for: Paf1c defects challenge the robustness of flower meristem termination in Arabidopsis thaliana
Source: Development. 2019 Oct 25;146(20):dev173377. doi: 10.1242/dev.173377 (PMC6826038; doi:10.1242/dev.173377)
Supplement: Supplementary information [file develop-146-173377-s1.pdf]

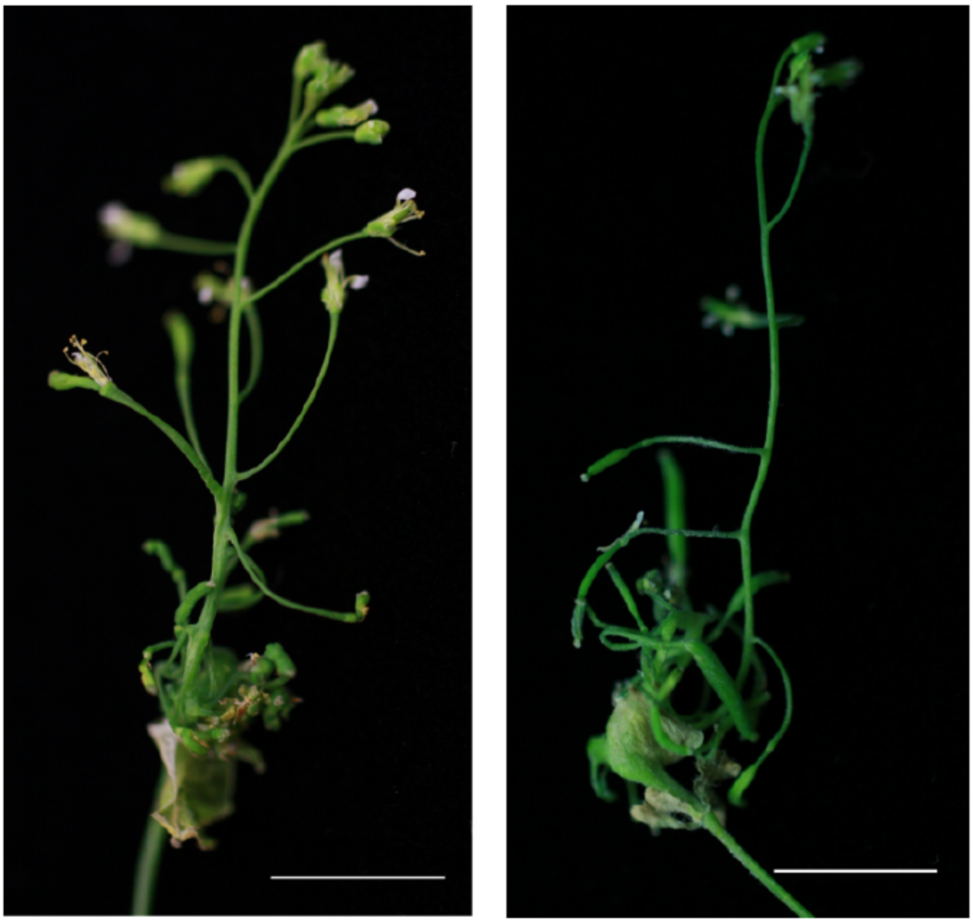

Fig. S1. Examples of the most severe indeterminacy phenotypes in *vip3-1* siliques.

Representative images of the most severe phenotypes in *vip3-1* flowers, displaying an inflorescence stem with siliques and flowers, emerging from a silique. Scale bar: 1 cm.

Development • Supplementary information

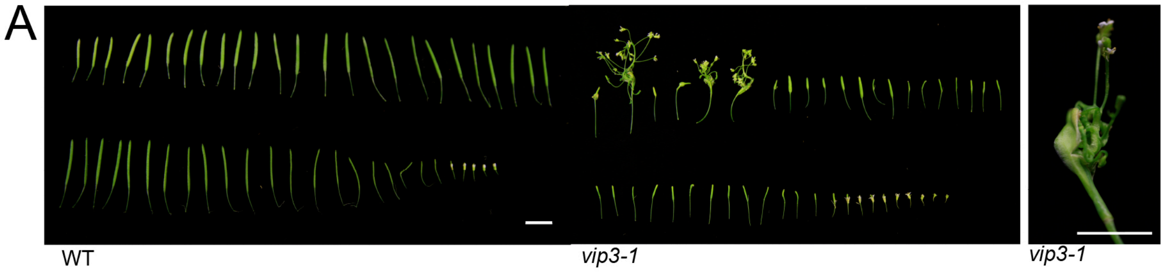

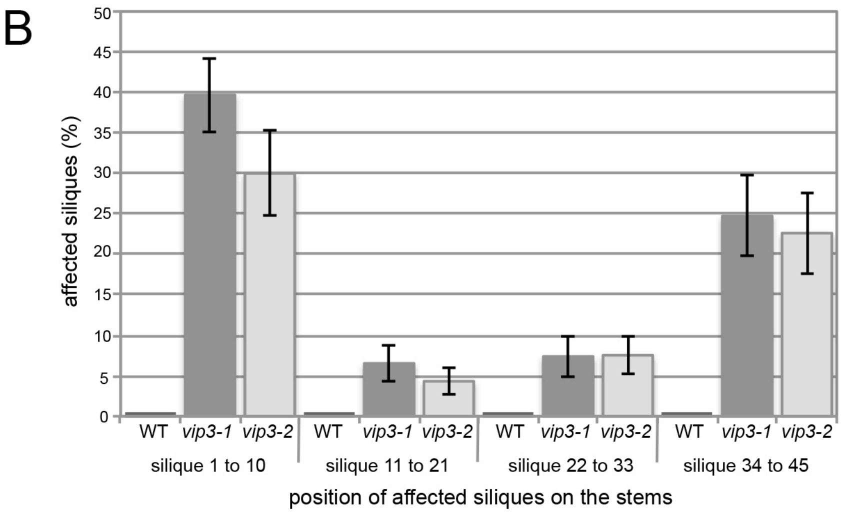

Fig. S2. *vip3* phenotype in short day then continuous light 21°C.

(A) Phenotype of WT (left panel) and *vip3-1* (middle panel) siliques, from plants grown in short day 21°C followed by continuous light 21°C conditions, harvested in a sequence of initiation along the stem. Scale bar : 1 cm. Right panel shows representative silique of the *vip3* displaying the indeterminacy phenotype. Scale bar : 5 mm. (B) Distribution (%) of affected siliques along the stems of the wild type (N=13), *vip3-1* (N=32) and *vip3-2* (N=21) grown in short day 21°C followed by continuous light 21°C condition (on average, 19% of *vip3-1* and 17% of *vip3-2* siliques displayed visible indeterminacy defects in these conditions).

Development • Supplementary information

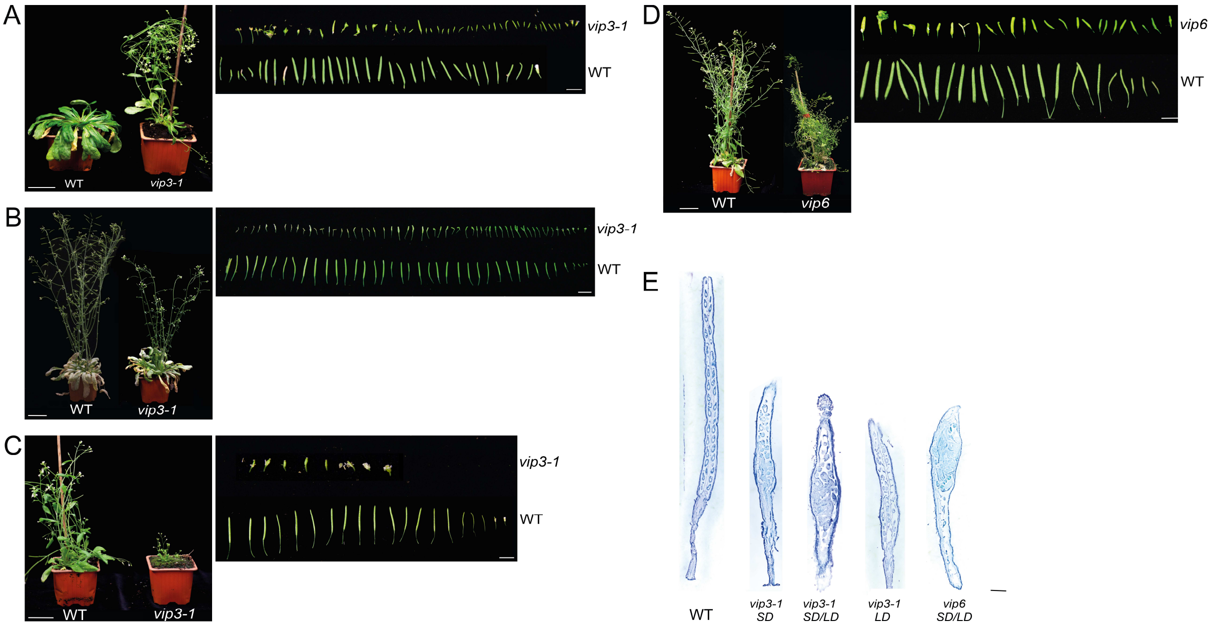

Fig. S3. Indeterminacy phenotype in different growth conditions and in different mutants of the Paf1 complex.

(A-C) Phenotypes of WT and *vip3-1* mutants grown in short day conditions (A, N=9 plants), in short then long day conditions (B, N=22 plants), and in long day conditions (C, N=22 plants). (D) Phenotype of WT and *vip6* mutant grown in short day 21°C followed by continuous light 16°C conditions displaying the indeterminacy phenotype (N=19 plants). For each condition, left panels display wild-type and *vip* adult plants, and right panels the siliques harvested in the order of their initiation along the stem. (E) Representative sections in young siliques, stained with toluidine blue, of *vip3-1*, in each culture condition, and *vip6* mutant, displaying the indeterminacy phenotype. Scale bars : 3 cm (A-D, left panels); 1 cm (A-D, right panels); 500 µm (E).

Development • Supplementary information

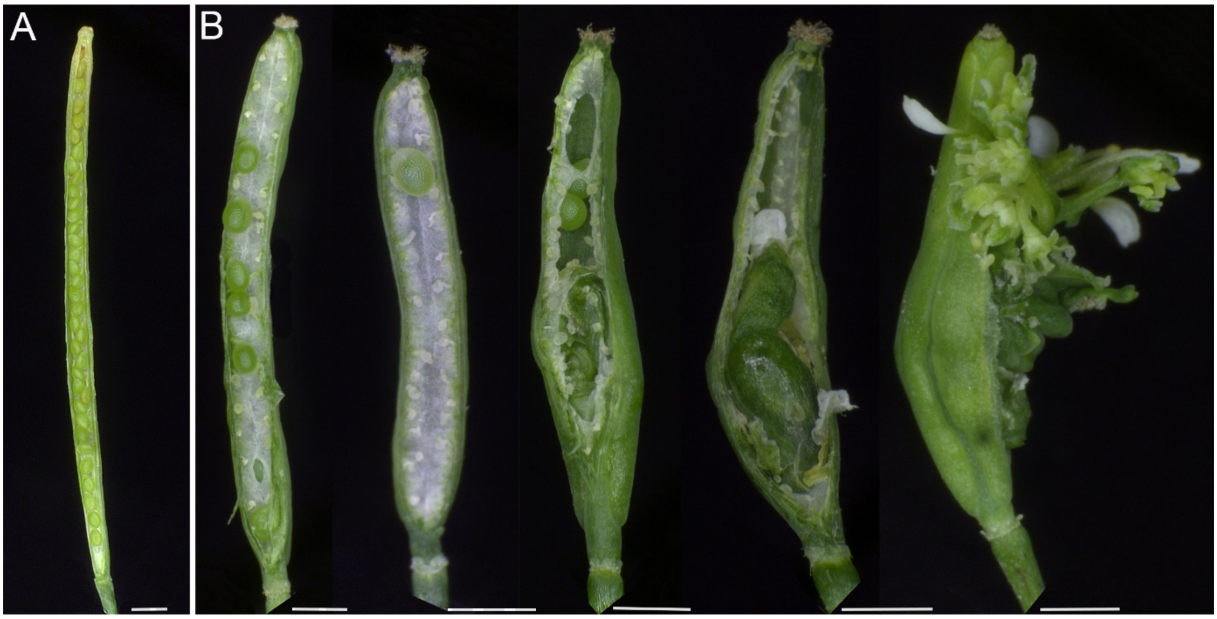

C	Number of ovules per silique		Number of aborted ovules per silique		Number of fertilized ovules per silique	
	WT	vip3-1	WT	vip3-1	WT	vip3-1
Mean	69,4	74,3	2,8	19,7	64,6	6,1
Standard deviation	3,9	18,2	0,9	11	3,7	6,8
Standard error of the mean	0,5	2,1	0,1	1,3	0,4	0,8
% compare to the total number of ovules per silique	-	-	4	26,5	93,1	8,2

Fig. S4. Proportion of aborted ovules and seeds in *vip3-1*.

(A-B) illustrates the range of phenotypes observed in *vip3-1* (B) compared to Col0 (A) (grown in short day and then in continuous light 16°C). *vip3-1* displays a strong and highly variable reduction of seed set in siliques showing no indeterminacy. In silique showing indeterminacy no or very few seeds usually develop. Bars = 500 µm. (C) Number of aborted ovules and seeds in *vip3-1* (N=73 siliques) and WT (N=70 siliques). The standard deviation reflects the variability of the original distribution. The standard error of the mean indicates the precision of estimated means (95% confidence interval).

Development • Supplementary information

A

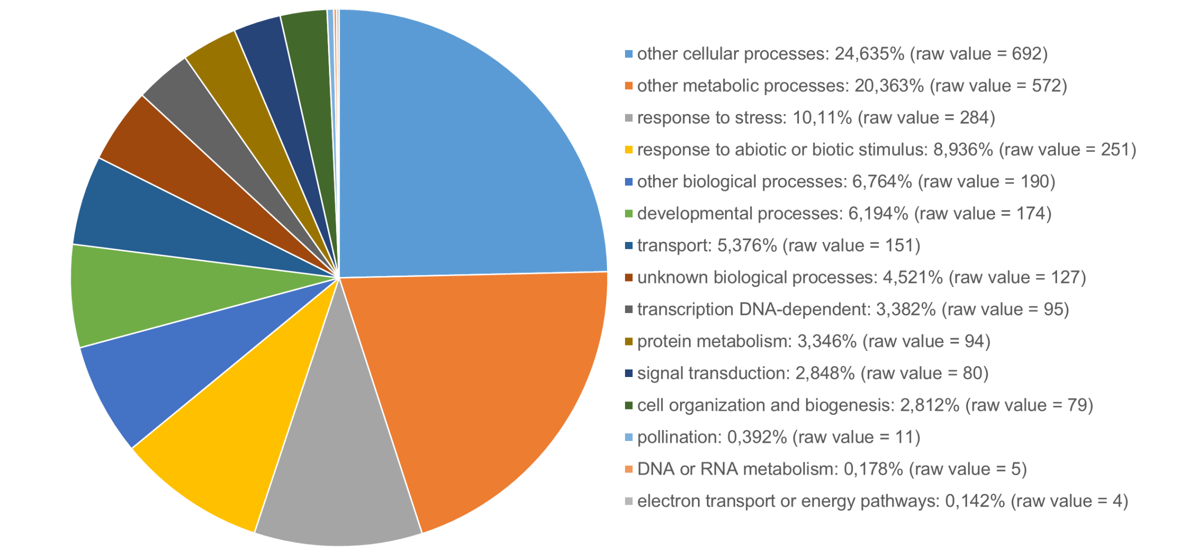

B

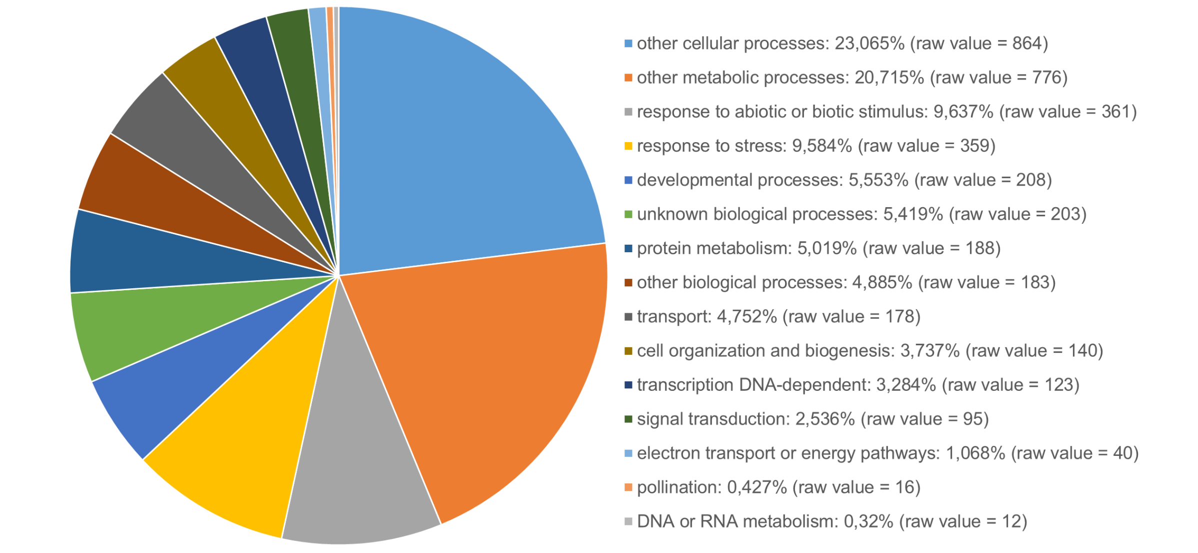

C

	gene_id	gene	log2(fold_change)	p_value
Downregulated	AT1G77080	FLM, FLOWERING LOCUS M / MAF1, MADS AFFECTING FLOWERING 1	-8,89394	5,00E-05
	AT5G65060	FCL3, FLOWERING LOCUS 3 / MAF3, MADS AFFECTING FLOWERING 3	-8,38456	0,0018
	AT5G10140	FLC, FLOWERING LOCUS C	-4,65699	5,00E-05
	AT5G65080	AGL68, AGAMOUS-LIKE 68 / MAF5, MADS AFFECTING FLOWERING 5	-4,15656	5,00E-05
	AT5G65050	AGL31, AGAMOUS-LIKE 31 / MAF2, MADS AFFECTING FLOWERING 2	-3,93607	5,00E-05
	AT5G38740	AGL77, AGAMOUS-LIKE 77	-3,33443	5,00E-05
	AT2G20825	ULT2, ULTRAPETALA2	-3,10298	5,00E-05
	AT5G27580	AGL89, AGAMOUS-LIKE 89	-2,67671	0,00405
	AT4G24540	AGL24, AGAMOUS-LIKE 24	-2,29325	5,00E-05
	AT4G27330	NZZ, NOZZLE / SPL, SPOROCYTELESS	-1,86166	0,00155
	AT5G60440	AGL62, AGAMOUS-LIKE 62	-1,59424	0,00795
	AT5G21150	AGO9, ARGONAUTE 9	-1,2134	5,00E-05
	AT5G57390	AIL5, AINTEGUMENTA-LIKE 5 / CHO1, CHOTTO 1 / EMK, EMBRYOMAKER / PLT5, PLETHORA 5	-0,866008	5,00E-05
	AT2G03060	AGL30, AGAMOUS-LIKE 30	-0,794353	0,001
	AT2G26440	PME12, PECTIN METHYLESTERASE 12	-0,709637	5,00E-05
Upregulated	AT3G20810	JMJ30 / JMJD5, JUMONJI DOMAIN CONTAINING 5	0,505522	0,00085
	AT4G37650	SGR7, SHOOT GRAVITROPISM 7 / SHR, SHORT ROOT	0,507952	0,00035
	AT2G33880	WOX9, WUSCHEL-RELATED HOMEOBOX 9	0,571996	0,00045
	AT2G34710	PHB, PHABULOSA / ATHB-14, ARABIDOPSIS THALIANA HOMEOBOX PROTEIN 14	0,586075	5,00E-05
	AT1G62360	STM, SHOOTMERISTEMLESS	0,589834	0,0001
	AT1G19850	MP, MONOPTEROS / ARF5, AUXIN RESPONSE FACTOR 5	0,608684	0,0019
	AT3G11050	FER2,- FERRITIN 2	0,652661	0,0039
	AT1G69770	CMT3, CHROMOMETHYLASE 3	0,707429	5,00E-05
	AT4G20270	BAM3, BARELY ANY MERISTEM 3	0,779995	5,00E-05
	AT5G62230	ERL1, ERECTA-LIKE 1	0,835578	5,00E-05
	AT5G53950	CUC2, CUP-SHAPED COTYLEDON 2	0,910426	5,00E-05
	AT5G11320	YUC4, YUCCA4	1,05308	5,00E-05
	AT2G45660	SOC1, SUPPRESSOR OF OVEREXPRESSION OF CO 1	1,0598	5,00E-05
	AT4G00150	HAM3, HAIRY MERISTEM 3, ATHAM3, HAIRY MERISTEM 3, HAM3, LOM3, LOST MERISTEMS 3, SCL6-IV	1,08428	5,00E-05
	AT1G68640	PAN; PERIANTHIA, TGA8, TGACG SEQUENCE-SPECIFIC BINDING PROTEIN 8	1,10476	5,00E-05
	AT3G15400	ATA20, ANTHER 20	2,05576	5,00E-05
	AT2G27250	CLV3, CLAVATA3	2,39061	5,00E-05
	AT1G75940	ATA27, BETA GLUCOSIDASE 20, BGLU20	2,50951	5,00E-05
	AT5G51870	AGL71, AGAMOUS-LIKE 71	2,85072	5,00E-05
	AT4G28395	ATA7, ARABIDOPSIS THALIANA ANTHER 7	3,11384	5,00E-05

D

	gene_id	gene	log2(fold_change)	p_value
Downregulated	AT2G14610	PR1; pathogenesis-related protein 1	-5,18392	5,00E-05
	AT5G59220	HAI1; PP2C protein (Clade A protein phosphatases type 2C)	-1,72227	5,00E-05
	AT4G34760	SAUR-like auxin-responsive protein family	-1,13968	5,00E-05
	AT1G08320	TGA9; bZIP transcription factor family protein	-0,969944	0,00355
	AT1G67710	ARR11; response regulator 11	-0,934393	0,00065
	AT3G23030	IAA2; indole-3-acetic acid inducible 2	-0,884945	0,0009
	AT4G34000	ABF3; abscisic acid responsive elements-binding factor 3	-0,857165	5,00E-05
	AT5G54510	DFL1; Auxin-responsive GH3 family protein	-0,799458	5,00E-05
	AT5G57560	TCH4; Xyloglucan endotransglucosylase/hydrolase family protein	-0,672642	5,00E-05
	AT3G23050	IAA7; indole-3-acetic acid 7	-0,628497	0,00325
	AT1G03430	AHP5; histidine-containing phosphotransfer factor 5	-0,590316	0,0007
	AT4G34750	SAUR-like auxin-responsive protein family	-0,579414	0,00535
Upregulated	AT1G80100	AHP6; histidine phosphotransfer protein 6	0,514231	0,0006
	AT2G22670	IAA8; indoleacetic acid-induced protein 8	0,537947	0,00075
	AT3G63010	GID1B; alpha/beta-Hydrolases superfamily protein	0,572849	0,00025
	AT1G28130	GH3.17; Auxin-responsive GH3 family protein	0,575311	5,00E-05
	AT5G46570	BSK2; BR-signaling kinase 2	0,576718	5,00E-05
	AT1G19850	MP; Transcriptional factor B3 family protein / auxin-responsive factor AUX/IAA-like protein	0,608684	0,0019
	AT1G51950	IAA18; indole-3-acetic acid inducible 18	0,628133	5,00E-05
	AT5G46790	PYL1; PYR1-like 1	0,646811	5,00E-05
	AT2G38120	AUX1; Transmembrane amino acid transporter family protein	0,691917	5,00E-05
	AT2G01570	RGA1; GRAS family transcription factor family protein	0,759926	5,00E-05
	AT1G45249	ABF2; abscisic acid responsive elements-binding factor 2	0,794846	5,00E-05
	AT1G19050	ARR7; response regulator 7	0,811842	5,00E-05
	AT2G38310	PYL4; PYR1-like 4	0,856148	5,00E-05
	AT4G27260	WES1; Auxin-responsive GH3 family protein	0,864813	5,00E-05
	AT1G72450	JAZ6; jasmonate-zim-domain protein 6	0,885443	5,00E-05
	AT1G17380	JAZ5; jasmonate-zim-domain protein 5	0,960683	5,00E-05
	AT4G33950	OST1; Protein kinase superfamily protein	1,02011	5,00E-05
	AT5G13220	JAZ10; jasmonate-zim-domain protein 10	1,03421	5,00E-05
	AT1G19180	JAZ1; jasmonate-zim-domain protein 1	1,04499	5,00E-05
	AT5G11320	YUC4, YUCCA4	1,05308	5,00E-05
	AT2G41310	RR3; response regulator 3	1,11732	5,00E-05
	AT1G77920	TGA7; bZIP transcription factor family protein	1,15091	0,00385
	AT5G17490	RGL3; RGA-like protein 3	1,15216	5,00E-05
	AT3G11410	PP2CA; protein phosphatase 2CA	1,21844	5,00E-05
	AT4G14550	IAA14; indole-3-acetic acid inducible 14	1,31153	0,0009
	AT1G04250	AXR3; AUX/IAA transcriptional regulator family protein	1,58048	0,0001
	AT3G21510	AHP1; histidine-containing phosphotransmitter 1	1,80767	0,00015
	AT1G77690	LAX3; like AUX1 3	2,1705	5,00E-05
	AT4G00880	SAUR-like auxin-responsive protein family	2,62316	5,00E-05
	AT5G13380	Auxin-responsive GH3 family protein	3,54665	5,00E-05
	AT2G46690	SAUR-like auxin-responsive protein family	3,63345	5,00E-05

Fig. S5. Differential gene expression in *vip3-1* vs. wild-type shoot apices.

(A, B) Gene ontology analysis: categories of genes involved in biological processes that are up-regulated (A) and down-regulated (B) in *vip3-1*. (C, D) Short list of genes involved in flowering and flower development (C) and signaling (D) pathways that are misexpressed in *vip3-1*. Genes that are down-regulated are highlighted in blue, and those that are up-regulated are highlighted in orange.

Development • Supplementary information

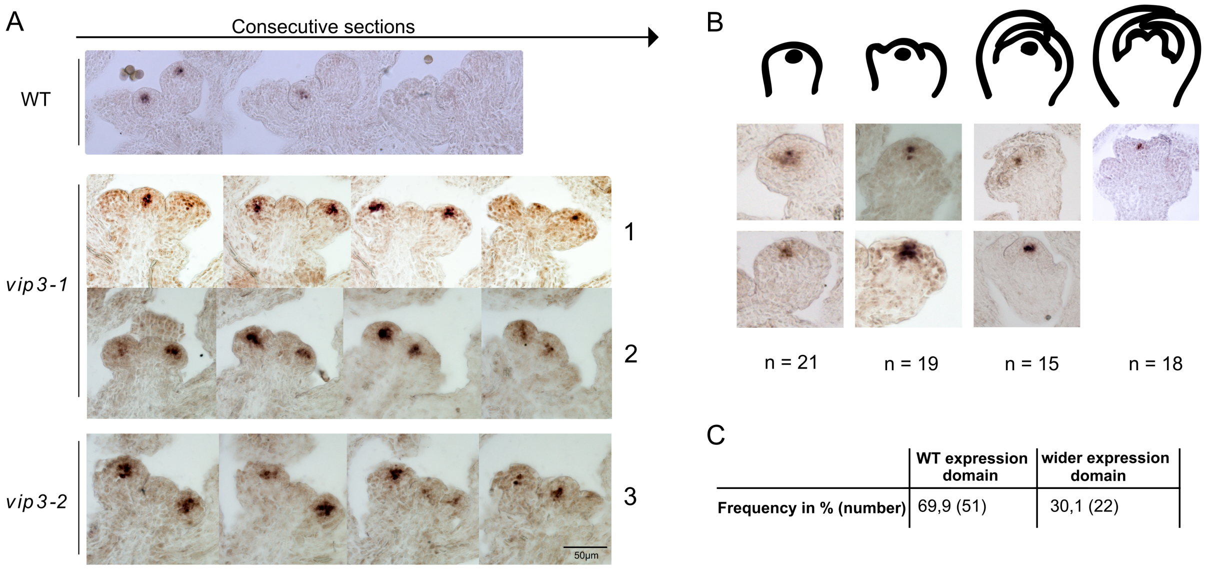

Fig. S6. Expression patterns of *WUS* in *vip3* flowers.

(A, B) *In situ* hybridization of *WUS* transcripts in wild-type (A, upper panel) and *vip3-1* (A, lower panel and B). (A) Consecutive sections on WT and 3 independent *vip3* (1-2: *vip3-1*; 3: *vip3-2*) apices showing an expansion of *WUS* expression domain in *vip3-1* floral meristems, when compared to wild type. (B) Representative patterns of *WUS* expression domain in *vip3-1* flower buds at four different developmental stages (as represented by schematic drawings). Plants were grown in short day then continuous light 16°C conditions (as in Fig. 1). Scale bar: 50 µm. (C) Number of flower meristems displaying a wild-type *WUS* expression domain and an enlarged *WUS* expression domain in *vip3-1*.

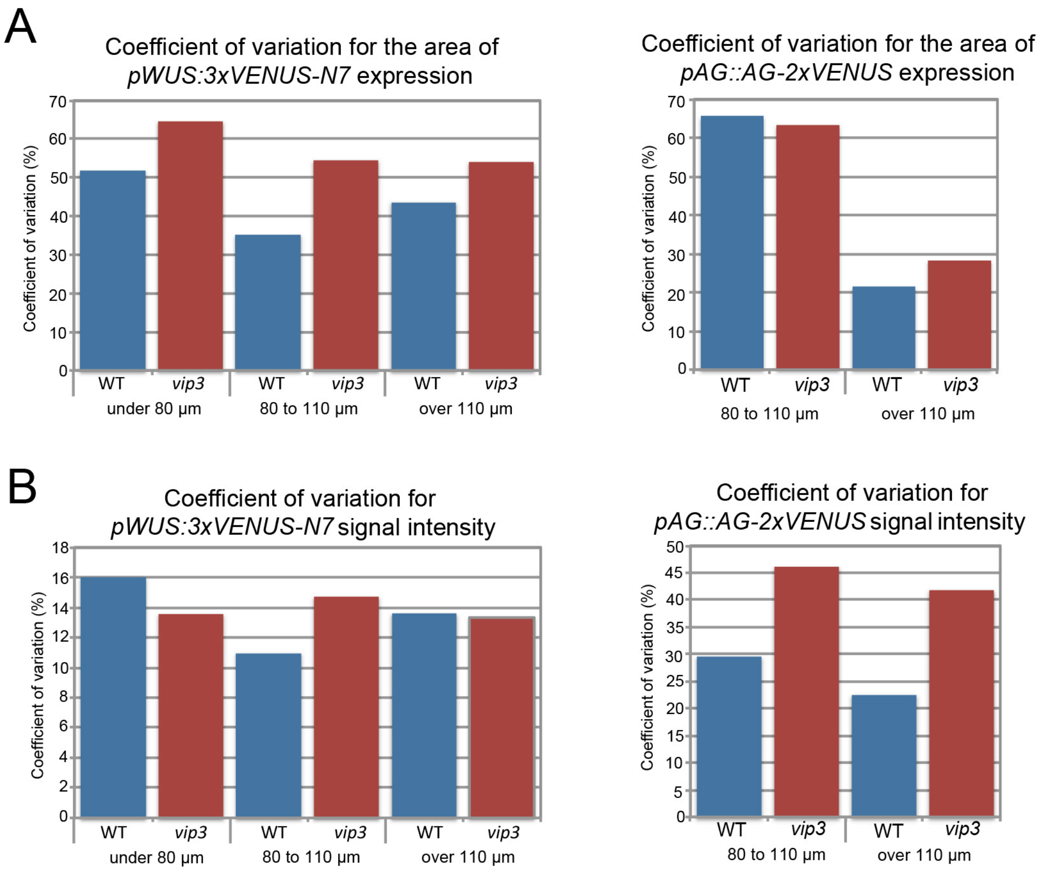

Fig. S7. Coefficient of variation for *WUS* and *AG* expression area and average intensity in *vip3* flowers

(A) Histograms displaying the coefficients of variation (%) for the area of *pWUS::3xVENUS-N7* (left) and *pAG::AG-2xVENUS* (right) expression in wild-type and *vip3-1* flowers. (B) Histograms displaying the coefficients of variation for the average fluorescence signal intensity of *pWUS::3xVENUS-N7* (left) and *pAG::AG-2xVENUS* (right) expression in wild-type and *vip3-1* flowers.

Development • Supplementary information

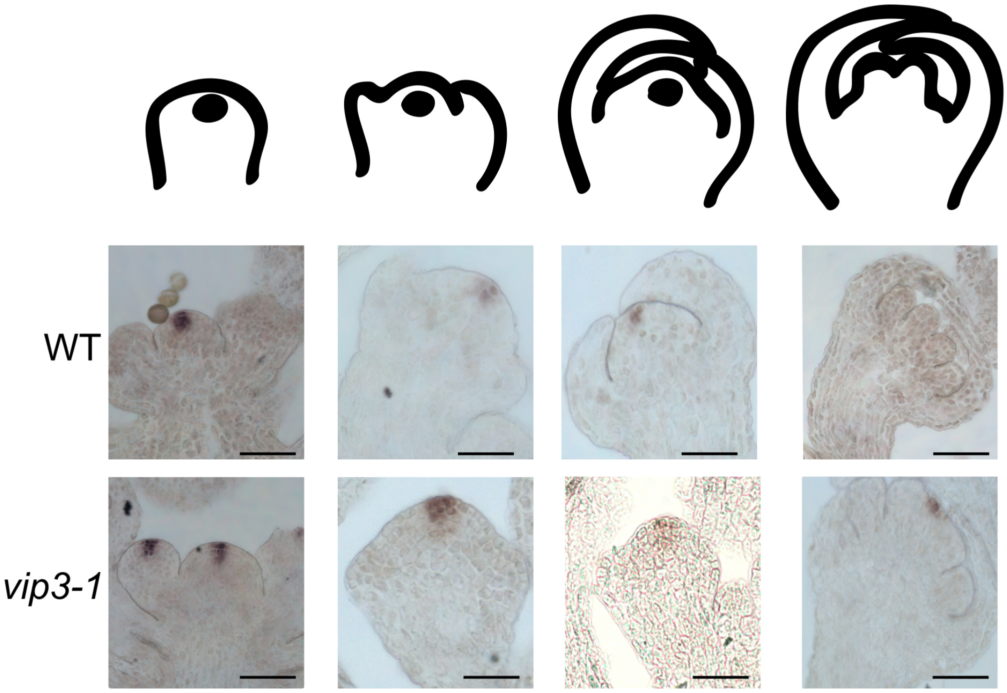

Figure S8. Expression patterns of *CLV3* in *vip3-1* flowers

In situ hybridization of *CLV3* transcripts in wild-type (A) and *vip3-1* (B). Plants were grown in short day then continuous light 16°C conditions (as in Figure 1). Scale bar = 50 µm.

Table S1. List of primers

Name	Sequence
Genotyping primers	
LBb1.3	ATTTTGCCGATTTCGGAAC
vip3-1 F	GACTGCAAGTACCACTTTCGC
vip3-1 R	TAATGGGAAACGACTTGCTTG
vip3-2 F	CTGACTGGATCTCTTGACGAGACG
vip3-2 R	GATACTCAGCAATTCCATATAGTACCCAAGC
Primers for *in situ* probes	
*WUS_in_situ*_F	CAACAAGTCCGGCTCTGGTG
*WUS_in_situ*_RT7	TGTAATACGACTCACTATAGGGCGGGAAGAGAGGAAGCGTACGTCG
*AG_in_situ*_F	ACGGCGTACCAATCGGAGCT
*AG_in _situ*_RT7	TGTAATACGACTCACTATAGGGCGTTGCAATGCCGCGACTTGG
CLV3_in_situ_F	ATGTCCGGTCCAGTTCAACAAC
CLV3_in_situ_RT7	TGTAATACGACTCACTATAGGGCGGTCAGGTCCCGAAGGAACA
Primers for *pAG::AG-2xVenus* construction	
pPD381	GTCCCCGGGAGTGATCCCTTCTCCAACACA
pPD413	AGTCCCGGGTAACTGGAGAGCGGTTTGGT
pPD441	AGTGGATCCGCAGCTGCCGCAGCTGCGATGGTGAGCAAGGGCGAG
pPD442	GTCTCTAGACTAGATAGATCTCTTGTACAGCTC

Development • Supplementary information
